# Clam shell waste recycling and valorization for sustainable Hg remediation

**DOI:** 10.1016/j.heliyon.2024.e35375

**Published:** 2024-07-27

**Authors:** Silvano Mignardi, Emanuele Tocci, Laura Medeghini

**Affiliations:** a Department of Earth Sciences, Sapienza University of Rome, P.le Aldo Moro, 5, 00185, Rome, Italy; b CIABC, Sapienza University of Rome, P.le Aldo Moro, 5, 00185, Rome, Italy

**Keywords:** Clam shell, Waste valorization, Hg^2+^ remediation, Hydroxyapatite, Value-added product

## Abstract

Shellfish aquaculture world production is constantly growing due to the increase in demand for seafood and reached over 18 million tons in 2022. The suitable management of the shell waste is one of the main environmental challenging issues as most of this waste is sent to landfills with emanation of foul odors, pathogens proliferation and reduction of available space. However, the conversion of this biowaste to new value-added materials could provide significant environmental and economic benefits. Clam shell waste was the starting material for the synthesis of hydroxyapatite (CSHAP) applied as an adsorbent for Hg^2+^ removal from aqueous solutions. Adsorption experiments were performed in batch using simulated wastewaters prepared from HgCl_2_ to investigate the effects of contact time and initial Hg^2+^ concentration on the removal process. Mineralogical composition, morphological features and elemental composition of CSHAP before and after the experiments were investigated by XRPD, SEM-EDS and FTIR analysis. The concentrations of Hg^2+^ and Ca^2+^ in the solutions were analyzed by ICP-AES. The adsorption kinetics of Hg^2+^ was simulated with the pseudo-first-order rate model, the pseudo-second order model and the intraparticle diffusion model. The results of the kinetics study showed that the Hg^2+^ adsorption followed the pseudo-second-order kinetics model and reached equilibrium within 40 min. The Langmuir model fitted the experimental results better than the Freundlich, Temkin and Dubinin-Radushkevic isotherm models, with a maximum adsorption capacity of 65.8 mg/g which is generally higher than other waste-derived adsorbents used for the removal of Hg^2+^ ions from water. The removal mechanism includes rapid surface complexation on CSHAP grains, followed by a slow incorporation of the Hg^2+^ ions in the crystalline structure. The results of this study could contribute to delineate a new research direction for a more sustainable management of clam shell biowaste.

## Highlights

• Clam waste shells were converted into hydroxyapatite (CSHAP).
• The adsorbent potential in removing Hg^2+^ from aqueous solutions was evaluated.
• CSHAP removal efficiency reached 94 % in 40 min.
• Hg^2+^ removal involves surface complexation and incorporation in the structure.
• The application of waste-derived adsorbent contributes to waste minimization.

## Graphical abstract

**Figure:**
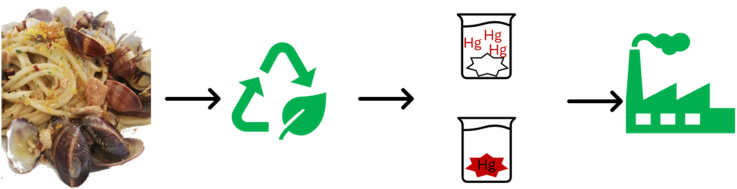

## 1 Introduction

The linear economic system based on consumption-production-disposal relies on the unsustainable exploitation of large quantities of energy, resources and materials. On the other hand, the circular economy system is recognized as a powerful integrative model designed to address the problems that our society is facing regarding the exhaustion of resources and environmental pollution. This economy model is rapidly affecting numerous segments of the economy, such as production, consumption and recycling [1]. At the basis of these concepts is the correct management of waste. Prevention, reduction and reuse must always be given priority. But when this is not possible, recycling takes priority [2,3].

A production sector facing a constant increase in the volume of waste is shellfish aquaculture due to the growing demand for seafood [4,5]. World production in 2022 was over 18 million tons [6] and Italy is among the top 10 countries in the world, with a production of over 100,000 tons per year (about 0.7 % of the total) [4,7]. In shellfish aquaculture, one of the key factors is the management of shells, which make up to 75 % of the total organism weight [4]. Most of this waste is sent to landfills, favoring the proliferation of pathogens, bad smell and reduction of available space [4,[8], [9], [10]]. However, considering that the shells are composed of 95–99.9 % of calcium carbonate [4], the ideal solution would be to recycle and reuse this waste as a raw material for the production of new materials, with significant environmental and economic benefits. The production of useful biomaterials starting from this food waste is a consolidated example of a circular, efficient, sustainable and economical process of waste management, from recycling to its reintegration into production cycles [11,12]. The use of calcium carbonate obtained from shell waste is the most suitable alternative to the use of calcium carbonate of geological origin for the production of biomaterials such as, for example, hydroxyapatite (HAP) for the sustainable treatment of toxic metal-polluted waters, also helping to reduce the impact of mining on natural reserves of phosphate rocks [11,[13], [14], [15], [16]].

Hydroxyapatite is a mineral with the formula Ca_10_(PO_4_)_6_(OH)_2_ belonging to the phosphate-calcium apatites and is widely used for the removal of toxic metals from polluted water and soil [17,18]. The main removal mechanisms are processes of adsorption, ion exchange, dissolution-precipitation and surface complexation [[19], [20], [21]]. HAP is very efficient in the immobilization of metals such as Cr, Pb, Cd, Ni, Zn, Cu, Co and Mn [22,23].

Toxic metal pollution of water and soils is a global environmental problem due to the carcinogenic properties of metals, bioaccumulation in organisms and the fact that they are not biodegradable [[24], [25], [26]]. Among toxic metals, mercury is one of the most dangerous pollutants worldwide, the main toxicological effects of which include neurological damage, paralysis, blindness and carcinogenesis [25]. Since the nineteenth century, with the intensification of mining activities and industrial production, the content of this element in water, soils and sediments has increased exponentially [27].

Mercury occurs in the environment in three main forms: elemental mercury (Hg^0^), inorganic mercury (Hg^2+^) and various organic forms, among which the most widespread is methylmercury (CH_3_Hg). It is estimated that between 250,000 and 1,000,000 tons of mercury are present in the environment [28]. The main natural sources are volcanic emissions, weathering of rocks, forest fires and evaporation from soil and water. In addition, the use of fossil fuels, mining activities, the treatment of minerals and the production of cement, are among the major anthropogenic sources of mercury and contribute two thirds of the total. In fact, it is estimated that anthropogenic activities would have led to the accumulation of about 86,000 tons of mercury in the soil and in the waters [28]. Unlike many toxic metals such as Cd, Pb, Cu and Zn, Hg removal processes using HAP have so far received little attention in the scientific literature [22,29].

To the best of our knowledge, this is the first experimental study to evaluate the adsorption capacity of Hg from aqueous solutions by HAP synthesized from clam waste shells (*Ruditapes philippinarum*) (CSHAP). Batch experiments were carried out to evaluate the effects of contact time and the initial concentration of Hg^2+^ on the efficiency of metal removal by the phosphate amendment. The Langmuir, Freundlich, Temkin and Dubinin-Radushkevic equations and various kinetic models were applied to evaluate the adsorption process. The results of this study showed that the transformation of a waste into an adsorbent suitable for toxic metal remediation can contribute also to the environmentally friendly management of clam shell waste.

## 2 Materials and methods

### 2.1 Materials

Analytical grade reagents from Sigma Aldrich Chemical Company were used in this study. Double distilled water was used for the solutions. Waste clam shells were collected from restaurants in Rome, Italy. The shells were rinsed with distilled water and oven-dried at 105 °C for 24 h. The crushed shells were sieved to obtain a homogeneous particle size of 100 μm and then stored in a desiccator. CSHAP was produced following the procedure previously described [20,23]: in a solution of HNO_3_ the shell powder was stirred (600–800 rpm) at room temperature for 1 h. The following reaction occurred:

(1) CaCO_3_ + 2HNO_3_ → Ca(NO_3_)_2(aq)_ + CO_2_ + H_2_O

The precipitation of CSHAP was obtained adding dropwise a H_3_PO_4_ solution to the Ca(NO_3_)_2(aq)_ solution, controlling the pH to 10 using a solution of NH_4_OH, according to the following reaction:

(2) 10Ca(NO_3_)_2_ + 6H_3_PO_4_ + 20NH_4_OH → Ca_10_(PO_4_)_6_(OH)_2_↓ + 20NH_4_NO_3_ + 18H_2_O

The suspension was maintained at boiling temperature for 1 h, then at room temperature for 24 h and finally filtered to collect the precipitated CSHAP.

### 2.2 Sorption experiments

A total of fifty batch sorption experiments were executed at 25 ± 2 °C. Hg^2+^ solutions were prepared dissolving HgCl_2_ in double distilled water. CSHAP (0.2 g) was mixed with Hg^2+^ solution (100 mL) in Nalgene beakers and stirred at 300 rpm, along with appropriate blank. The specific experimental setup was optimized based on the results of our preliminary kinetics experiments.

For the kinetics study, solutions containing 100 mg/L of Hg were used, collecting samples at different contact time in the range 0–180 min and filtered using 0.20 μm Nucleopore polycarbonate membrane filters. In subsequent sorption experiments optimized parameters were used. The experiments were conducted using Hg solutions at metal concentrations in the range 50–500 mg/L, this range being relevant for polluted waters derived from industrial or mining activities [30,31]. For a detailed investigation of the removal mechanism ten experiments were extended up to 12 weeks. The pH of the solutions was measured before and after the interaction period; however, no pH adjustment was applied during the sorption experiments to mimic the conditions of industrial wastewater treatment, where pH control is often unnecessary or difficult to obtain. Moreover, a back-ground electrolyte was not used. All the experiments were performed in duplicates to ensure accuracy, reliability and reproducibility of the collected data.

### 2.3 Characterization

X-ray powder diffraction (XRPD) analyses of CSHAP before and after Hg sorption were carried out on a parallel-beam Bruker AXS D8 Advance, operating in transmission in θ-θ geometry (Department of Earth Sciences, Sapienza University of Rome, Italy). The instrument is fitted with an incident-beam Göbel mirror, a position-sensitive detector (PSD) VÅNTEC-1 set to a 6° 2θ aperture, and a prototype of capillary heating chamber. Data were collected in step-scan mode in the 4–80° 2θ angular range (CuKα), using a step size of 0.0219° 2θ and a counting time of 10 s.

A FEI-Quanta 400 scanning electron microscope (SEM-EDS) operating at 20 kV, with X-ray energy-dispersive spectroscopy (Department of Earth Sciences, Sapienza University of Rome, Italy) was used to investigate the morphological features and the chemical composition of the samples.

FTIR spectra of CSHAP reacted at different Hg concentrations (50, 100, 150, 300 and 500 mg/L) after 3 weeks were analyzed by Fourier transform infrared spectroscopic (FTIR). Each sample was deposited onto a diamond compression cell and analyzed in transmission mode by a Thermo Nicolet iS50 FTIR spectrometer coupled with a Continuum IR microscope. The analyses were performed using a 15× objective, the background was acquired on the diamond cell close to the sample before each spectrum, and a minimum of three points have been collected for each sample. The spectra were acquired in the range 4000–650 cm^−1^, at the spectral resolution of 8 cm^−1^ and 40 scans.

The concentrations of Hg^2+^ and Ca^2+^ in the solutions after the sorption experiments were analyzed by inductively coupled plasma atomic emission spectrometry (ICP-AES) with a Varian Vista RL CCD Simultaneous ICP-AES spectrometer (Department of Earth Sciences, Sapienza University of Rome, Italy). Analytical detection limits for Hg^2+^ and Ca^2+^ were 0.04 and 0.03 mg/L respectively and analytical errors were estimated in the order of 3 %.

The Hg removal efficiency (*%R*) and the adsorption capacity (*q*_*e*_) were calculated with the following equations [32]:

(1a) *%R* = [(*C*_*0*_ – *C*_*e*_)/*C*_*0*_] × 100

(2b) *q*_*e*_ = (*C*_*0*_ – *C*_*e*_) × *V*/*M*

where *q*_*e*_ is the amount of Hg removed from the solution per gram of CSHAP (mg/g), *C*_*0*_ and *C*_*e*_ are initial and final concentrations after equilibrium (mg/L), *V* is the volume of the solution (L) and *M* the mass of CSHAP (g).

During the sorption experiments the change in the concentration of hydrogen ions was measured using a pH 510 Eutec pH-meter.

### 2.4 Adsorption isotherms

The experimental data at equilibrium was fitted using the models of Langmuir [33], Freundlich [34], Temkin [35] and Dubinin-Radushkevich [36] which are described in Table 1.

**Table 1:** Adsorption models used in this study and relative parameters.

**Isotherm**	**Equation**	**Parameters**
**Langmuir**	$\frac{C\text{e}}{q\text{e}}=\frac{1}{\mathrm{bq}\max}+\frac{Ce}{q\max}$	*C*_*e*_ (mg/L) = the metal concentration at equilibrium *q*_*e*_ (mg/g) = the amount of metal adsorbed at equilibrium *q*_max_ (mg/g) = the maximum adsorption capacity *b* (L/mg) = the Langmuir adsorption constant
**Freundlich**	log*q*_e_ = log*K*_F_ + $\frac{1}{n}$ log*C*_e_	*K*_*F*_ (mg/g) = the Freundlich constant related to adsorption capacity *n* (g/L) = the Freundlich constant related to adsorption intensity
**Temkin**	$qe=\frac{RT}{bT}\ln\;KT+\frac{RT}{bT}\ln\;Ce$	*R* (8.314 J/mol/K) = the universal gas constant *T* (K) = the absolute temperature *b*_*T*_ (J/mol) = the Temkin constant related to the heat of adsorption *K*_*T*_ (L/g) = the Temkin constant related to the equilibrium binding energy
**Dubinin-Radushkevich**	ln*q*_e_ = ln*X*_m_ - *βε*^2^ *ε* = *RT*ln(1 + 1/*C*_*e*_) *E* = $\frac{1}{\surd 2\beta }$	*X*_*m*_ (mg/g) = the theoretical saturation adsorption capacity *β* (mol^2^/J^2^) = the activity coefficient related to mean adsorption energy *ε* = the Polanyi potential *E* (kJ/mol) = the mean adsorption energy
**Pseudo-first-order**	$\log\left(qe-qt\right)=\log\;qe-\frac{k1}{2.303}t$	*q*_*t*_ (mg/g) = the amount of metal adsorbed at time *t* *t* (min) = contact time *k*_*1*_ (1/min) = the pseudo-first-order rate constant of adsorption
**Pseudo-second-order**	$\frac{t}{qt}=\frac{t}{qe}+\frac{1}{k2qe2\;}$	*k*_*2*_ (g/mg min) = the pseudo-second-order rate constant of adsorption
**Intraparticle diffusion**	*q*_t_ = *k*_i_*t*^0.5^ + *I*	*k*_*i*_ (mg/g min^0.5^) = the intraparticle diffusion rate constant *I* (mg/g) = provides information about the thickness of the boundary layer

The Langmuir isotherm model, one of the most used for quantifying adsorption capacity of various adsorbents, is based on single-layer coverage on the surface of the adsorbent and no interaction between the adsorbed molecules [37]. The Langmuir parameters can be used to calculate the constant *R*_*L*_ which provides information about the affinity between adsorbent and adsorbate and can be expressed as:

(3) $$R_{L}=\frac{1}{1+\mathrm{bCi}}$$

where *C*_*i*_ (mg/L) is the initial concentration of Hg^2+^ ions. The *R*_*L*_ values suggest the type of adsorption: irreversible (*R*_*L*_ = 0), favorable (0 < *R*_*L*_ < 1), linear (*R*_*L*_ = 1) or unfavorable (*R*_*L*_ > 1) [38].

The Freundlich isotherm model is applicable to multilayer adsorption on heterogeneous surfaces, with interaction between the adsorbed molecules [37]. The linearity of adsorption is indicated by the value of the Freundlich *n* constant: linear (*n* = 1); the adsorption is a chemical (*n* < 1) or physical (*n* > 1) process [39].

The Temkin isotherm model assumes a linear decrease of the heat of adsorption with the coverage of the adsorbate. The model ignores the very high and low amount of concentrations [37].

The Dubinin–Radushkevich (D-R) isotherm model is based on the assumption that a relationship between adsorption and the volume of the adsorbent pores occurs. Commonly, it is applied to describe physical or chemical adsorption [37,38]. The values of the mean adsorption energy *E* (kJ/mol) provide information about the nature of adsorption process: values in the range 8–16 kJ/mol suggest that the adsorption occurs through ion-exchange, values of *E* < 8 kJ/mol indicate that the adsorption process is physical, values of *E* > 16 kJ/mol suggest that the adsorption is chemical [39].

### 2.5 Adsorption kinetics

The adsorption kinetics of Hg^2+^ was simulated with the Lagergren pseudo-first-order rate model [40], the pseudo-second order model [41] and the intraparticle diffusion model [42] which are reported in Table 1.

The pseudo-first-order rate model assumes that the adsorption takes place exclusively at specific sites and does not involve any interaction between the absorbed ions. The maximum adsorption corresponds to a monolayer on the surface of the adsorbent [43].

The basic assumption of the pseudo-second order model is that the metal adsorption can be described by a second-order rate equation [43].

The intraparticle diffusion model assumes that the film diffusion can be overlooked and the rate-limiting step is intraparticle diffusion. Then the adsorbate uptake is proportional to the square root of time [42].

## 3 Results and discussion

### 3.1 CSHAP characterization

**Fig. 1:**
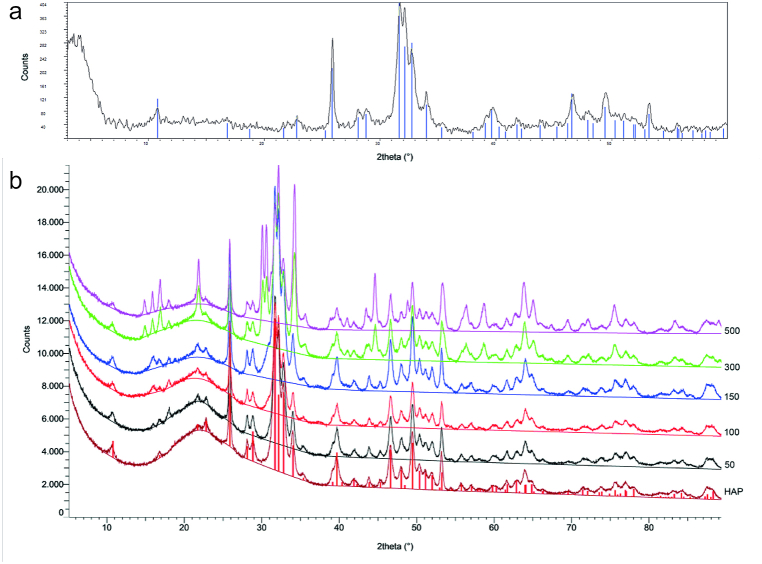
XRPD patterns of CSHAP before (a) and after (b) the interaction for 3 weeks with solutions containing 50, 100, 150, 300 and 500 mg/L Hg. The corresponding crystallographic diffraction lines of standards are plotted for comparison purposes.

The XRPD patterns of the starting CSHAP are in agreement with crystalline HAP (JCPDS card no. 09–0432) (Fig. 1a). After Hg adsorption for 3 weeks, the XRPD patterns are different for the various Hg concentrations (Fig. 1b). In particular, the variations of 2θ indicate a gradual expansion of the structure of the CSHAP unit cell, suggesting a progressive entry of Hg^2+^ ions into the crystal structure of the mineral. This finding agrees with those of previous studies [44], suggesting a slow mechanism of incorporation of Hg^2+^ ions into the crystalline structure of the phosphatic adsorbent after the fast surface complexation on the CSHAP grains in the first step of the process. Furthermore, we observe the appearance of better defined and higher intensity peaks (for example at about 70° and 83/84° 2θ) in the 300 and 500 mg/L patterns. However, despite the evidence of a principle of modification of the crystalline structure of the phosphate amendment, it was not possible to verify the actual formation of a new crystalline phase.

**Fig. 2:**
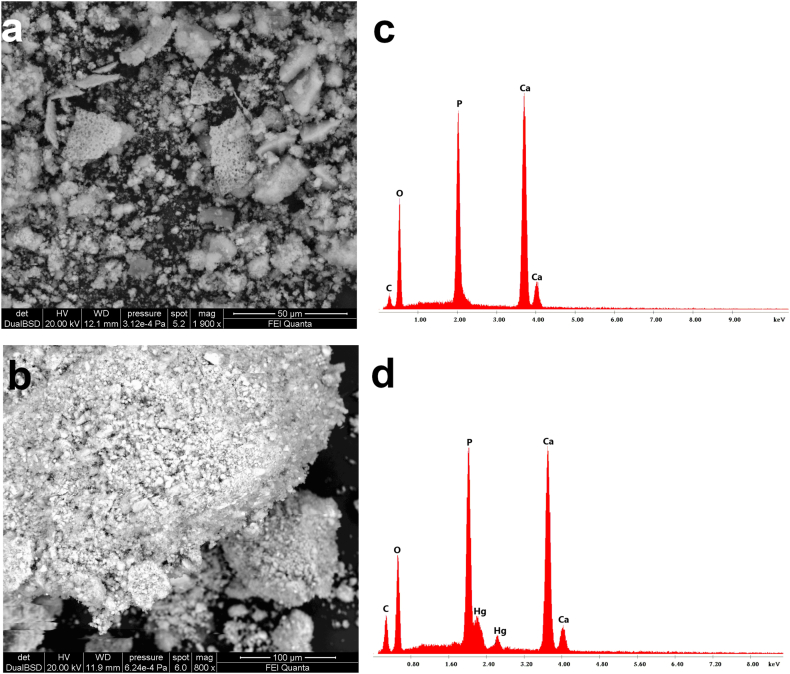
SEM images of CSHAP grains (a) before and (b) after Hg removal. EDS spectra of the (c) starting CSHAP particles showing peaks of Ca, P and O and (d) after Hg removal showing Hg peaks.

**Fig. 3:**
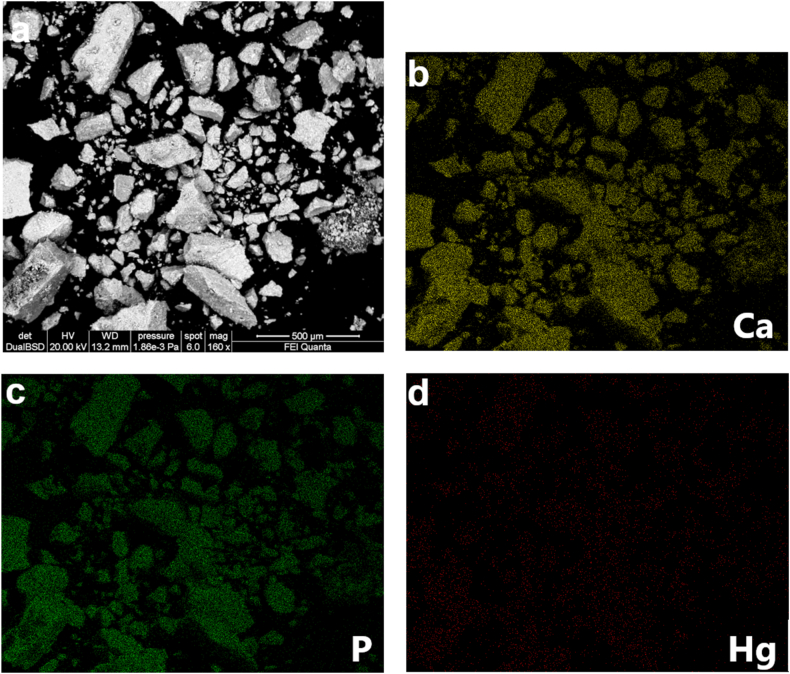
SEM image (BSE) of CSHAP after Hg^2+^ removal (a), EDS mapping dot analysis for Ca, P and Hg (b–d).

SEM analysis has been used to examine the morphological characteristics and external surface texture of CSHAP grains before and after Hg removal (Fig. 2). Before adsorption, CSHAP particles are clumped together showing a sheet or flake-like structure with sizes ranging from 20 to 30 μm (Fig. 2a). After the removal of Hg, the grains exhibit a smooth and uniform morphology (Fig. 2b) indicating that dissolution-precipitation processes play a significant role in the removal mechanism. The EDS spectra of the starting CSHAP particles show peaks of Ca, P and O (Fig. 2c). Upon interaction with Hg solutions strong Hg peaks appear in the spectra (Fig. 2d). EDS dot maps show the distribution of Ca, P and Hg in CSHAP grains (Fig. 3a–d). Ca and P are distributed on the entire surface of the grains, while Hg is abundantly and uniformly distributed on the CSHAP particles.

**Fig. 4:**
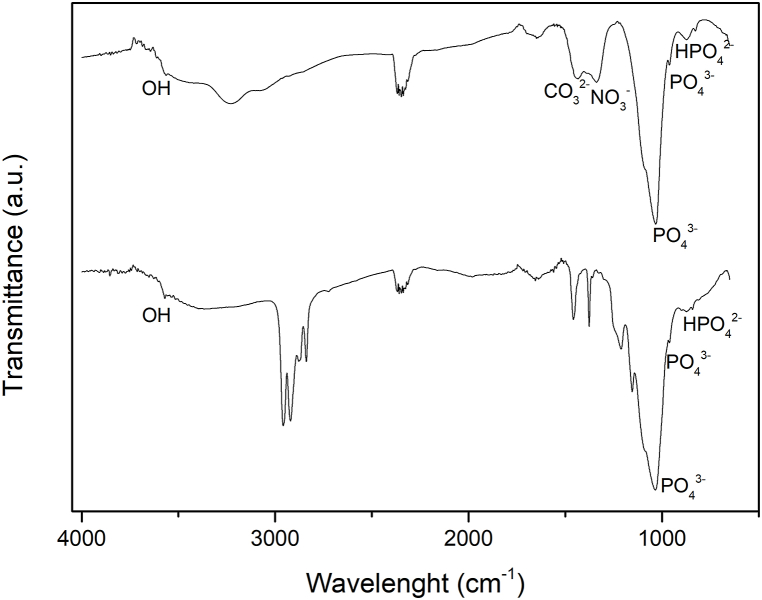
FTIR spectra of synthetized CSHAP and CSHAP reacted with 100 mg/L Hg^2+^ after 3 weeks. Phosphate, carbonate and nitrate groups have been labeled in the spectrum. Absorption bands at 1379, 1458, 2838, 2873, 2920 and 2958 cm^−1^ are due to the membrane filters.

FTIR spectra of synthetized CSHAP and that at contact of Hg show the characteristic bands of phosphate groups (Fig. 4). Particularly, bands at about 960 cm^−1^ are due to phosphate symmetrical stretching or *ν1*; whereas phosphate asymmetric stretching or *ν3* mode is identified by the bands between 1030 and 1100 cm^−1^ [45]. The band at 877 cm^−1^ was ascribed to HPO_4_^2−^ [46], which characterizes non-stoichiometric HAP deficient of Ca, however the broad shape of this band could hide a vibrational frequency of carbonate ions. The doublet around 1440 and 1650 cm^−1^ (along with that hidden at 873 cm^−1^) can be attributed to carbonate ions substituted in apatite structure [47]. The hydroxyl stretching, at about 3568 cm^−1^ is reconnected to intermolecular H bonds as well as that broad absorption band between 3000 and 3500 cm^−1^ [48]. Finally, the bands at about 820 and 1340 cm^−1^ were attributed to nitrate groups probably from synthesis residuals. CSHAP samples reacted at different Hg concentrations after 3 weeks show similar absorption bands (Fig. 4). The composition of the reacted sample is not homogeneous, indeed spectra collected in different points show different intensity of the same bands. Phosphate and OH bands remain, whereas that of carbonate at 1458 cm^−1^ disappears [49]. At higher Hg concentrations the samples were analyzed at 4, 8 and 12 weeks. Also in this case, spectra collected in different points show different intensity of the same bands, confirming again a not-homogeneous composition. The composition is the same of those previously reported, highlighting no changes in the structure of the compound.

### 3.2 Effect of contact time on adsorption

**Fig. 5:**
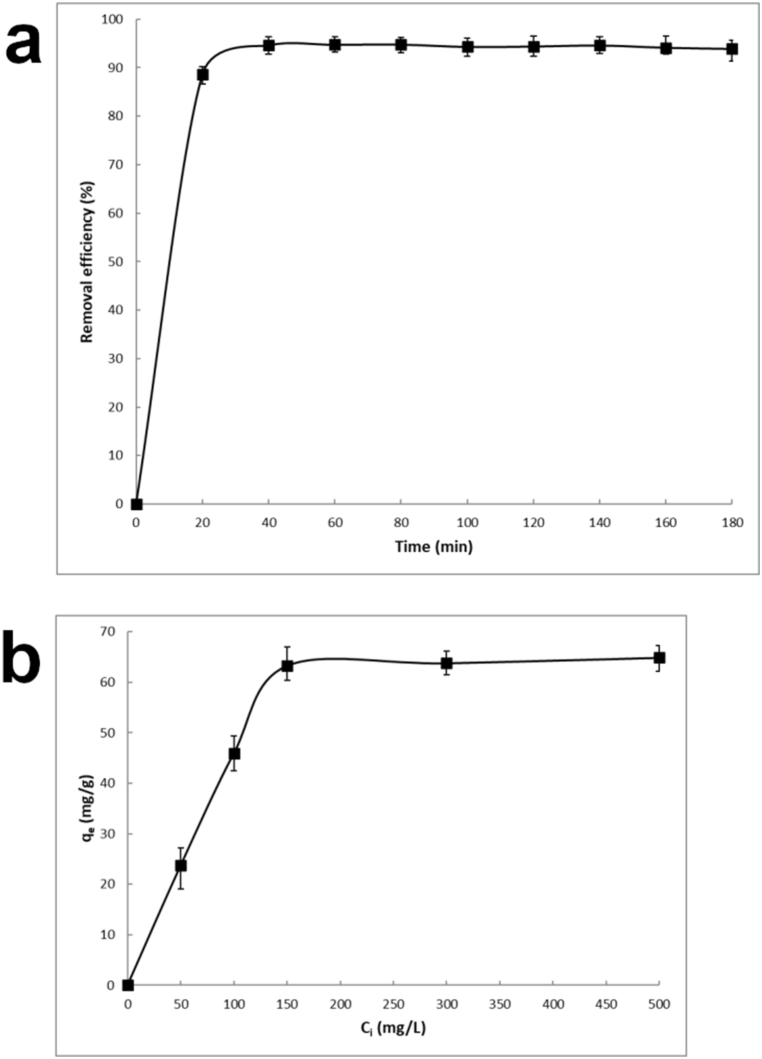
Effect of contact time (a) ([Hg^2+^] = 100 mg/L, CSHAP = 0.2 g, *T* = 25 ± 2 °C) and initial metal concentration (b) ([Hg^2+^] = 50–500 mg/L, CSHAP = 0.2 g, *T* = 25 ± 2 °C) on the removal of Hg^2+^ by CSHAP.

The Hg removal efficiency (eq. (1)) of CSHAP rapidly increases with contact time, reaching equilibrium in about 40 min with *%R* of 94.6 % (Fig. 5a). The removal of Hg^2+^ ions occurs in two phases: an initial rapid stage (first 40 min) followed by a slow stage until equilibrium is reached. The rapid initial Hg^2+^ uptake was determined by the numerous available vacant active sites on the surface of CSHAP particles and the high concentration gradient between Hg^2+^ in solution and metal ions on the CSHAP surface [50]. The slower removal efficiency in the final stage (40–180 min) resulted from the reduced concentration of metal ions in the solution and the decreased availability of active sites on the CSHAP particles.

### 3.3 Effect of initial Hg^2+^ concentration on adsorption

Increasing the initial Hg^2+^ concentration resulted in a higher amount of adsorbed metal ions (eq. (2)) until the equilibrium was reached (Fig. 5b). Thereafter, as all the active sites on CSHAP surface were occupied by Hg^2+^ ions, the adsorption of Hg^2+^ seems to no longer depend on the initial metal concentration. The removal of Hg^2+^ was accompanied by an increase in the concentrations of Ca^2+^ in the solutions. However, the weak correlation (*R*^*2*^ = 0.4254) between the amount of Hg^2+^ removed and the that of Ca^2+^ released in the solutions and the Hg/Ca molar ratios lower than 1 indicates that ion exchange only partially contributed to the overall removal mechanism, along with rapid surface adsorption of Hg^2+^ ions on the surface of CSHAP grains as reported in previous studies [51,52]. In this view, the results suggest a two-stage process for Hg adsorption. The first stage involves the rapid surface complexation on the CSHAP adsorbent, followed by the partial ion exchange with Ca ions within the crystalline structure during the second stage.

### 3.4 Adsorption study

**Fig. 6:**
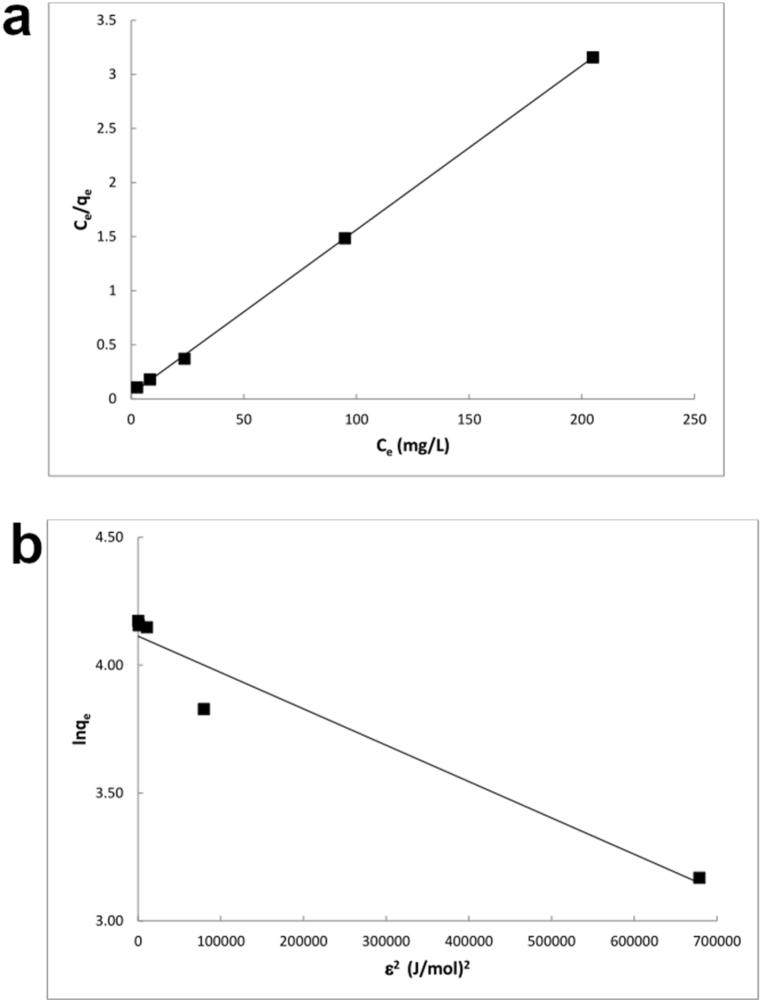
Adsorption isotherms for Hg^2+^ adsorption onto CSHAP; (a) Langmuir, (b) D-R models.

The experimental data fit better the Langmuir and D-R isotherm models (*R*^*2*^ = 0.9998 and 0.9488, respectively) than the Freundlich (*R*^*2*^ = 0.7501) and Temkin (*R*^*2*^ = 0.8030) models (Table 2, Fig. 6a and b). The strong fitting of the Langmuir model indicates that Hg^*2+*^ ions formed a homogeneous monolayer coverage on CSHAP particles. This is further corroborated by the close match between the *q*_*m,cal*_ value of the Langmuir model (65.8 mg/g) and the q_*m,exp*_ value (64.9 mg/g). The differences in the maximum adsorption capacity between the Langmuir and D-R models depend on the different definition of the maximum adsorption capacity [53]. Indeed, the value of the D-R model is lower than that of the Langmuir model as *X*_*m*_ represents the maximum adsorption capacity at the total specific micropore volume of the adsorbent, whereas *q*_*m*_ refers to monolayer coverage [54].

**Table 2:** Langmuir, Freundlich, Temkin and Dubinin-Radushkevich models parameters for the adsorption of Hg^2+^ on CSHAP.

	Langmuir			Freundlich			Temkin			Dubinin-Radushkevich			
*q*_m,exp_ (mg/g)	*q*_m,cal_ (mg/g)	*b* (L/mg)	*R*^2^	*K*_F_ (mg/g)	n (g/L)	*R*^2^	*b*_T_ (J/mol)	*K*_T_ (L/g)	*R*^2^	*X*_m,cal_ (mg/g)	*b* (mol^2^/kJ^2^)	*E* (kJ/mol)	*R*^2^
64.9	65.8	0.3227	0.9998	25.0207	4.7687	0.7501	276.5147	13.7894	0.803	61.1359	1.0∙10^−6^	707.11	0.9488

Information of the favorable adsorption process of Hg^2+^ ions by CSHAP is provided also by the values of the separation factor *R*_*L*_ (eq. (3)) ranging from 0.006 to 0.058 [39]. In addition, the values of the Freundlich constant *n* between 1 and 10 suggest favorable adsorption and high affinity between Hg^2+^ ions and CSHAP [38].

The value of the mean adsorption energy *E* (707.11 kJ/mol) higher than 16 kJ/mol indicates that chemisorption is the dominant mechanism [55].

The better fitting of Langmuir and D-R models than the Freundlich and Temkin suggests a uniform binding energy on the CSHAP surface, with Hg^2+^ ions not interacting or competing with each other.

The maximum adsorption capacity *q*_*m*_ of CSHAP has been compared with that of various waste-derived adsorbents used for the removal of Hg^2+^ ions from water (Table 3). Although different experimental conditions make difficult direct comparisons, the results show that the proposed adsorbent has a remarkable Hg^2+^removal capacity. Its primary advantage is the abundance of the starting material and the simplicity of the preparation method.

**Table 3:** Comparison of Hg^2+^ adsorption capacities for various waste-derived adsorbents.

Adsorbents	*q*_*m*_ (mg/g)	Reference
Candlenut activated charcoal	214.36	[56]
Corn straw biochar	269.08	[57]
Camel bone charcoal	28.24	[58]
Sugar cane bagasse	13.60	[59]
Rice husk-activated carbon	55.87	[60]
Peach stone activated carbon	59.50	[61]
Fish scales HAP	227.27	[62]
CSHAP	65.8	This study

### 3.5 Kinetic study

**Fig. 7:**
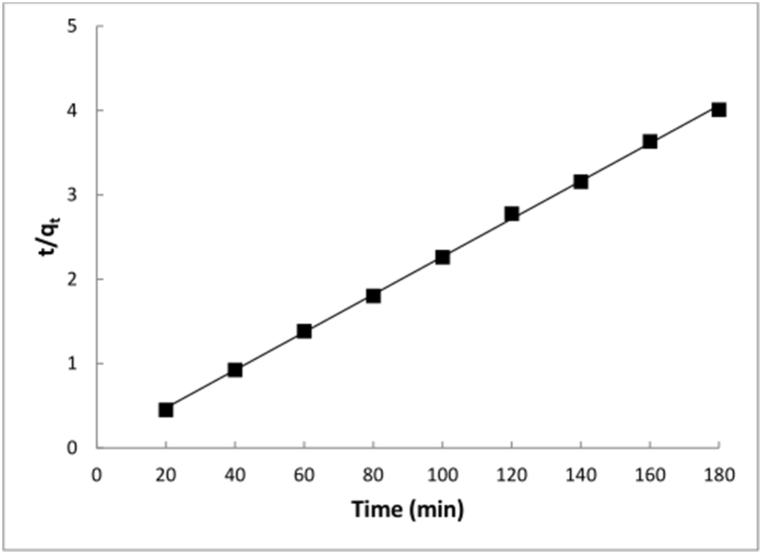
Pseudo-second-order kinetics for adsorption of Hg^2+^ onto CSHAP.

The pseudo-second-order model well described the adsorption of Hg^2+^ onto CSHAP (*R*_*2*_ = 0.9994) (Table 4, Fig. 7), with the *q*_*e,cal*_ value closely matching the experimental value (46.0 and 47.2 mg/g, respectively). This suggests that the overall adsorption process involves chemisorption, with valence forces involving electron sharing or exchange between adsorbent and adsorbate [39].

**Table 4:** Kinetics parameters of pseudo-first-order, pseudo-second-order and intraparticle diffusion models for Hg^2+^ adsorption on CSHAP.

	Pseudo-first-order model			Pseudo-second-order model				Intraparticle diffusion model		
*q*_e,exp_ (mg/g)	*q*_e,cal_ (mg/g)	*k*_1_ (1/min)	*R*^2^	*q*_e,cal_ (mg/g)	*k*_2_ (g/mg min)	*h*	*R*^2^	*k*_i_ (mg/g min^0.5^)	*I*	*R*^2^
47.2	6.8	0.0106	0.3760	46.0	0.0182	36.200	0.9994	0.0743	43.27	0.1354

The kinetics results were also analyzed using the intraparticle diffusion model. However, the very low correlation coefficient (*R*^*2*^ = 0.1354) indicates that this model does not adequately describe the Hg^2+^ adsorption process, implying that internal diffusion was weak during the adsorption process. In this view, other mechanisms such as surface complexation and ion exchange played significant roles in the Hg^2+^ adsorption process.

### 3.6 Current strategies for shell waste valorization

Today valorization of waste is a fundamental concept of circular economy, from environmental, social and economic point of view. In this view, mollusk shell waste can be given value following different approaches including, for example, the conversion to calcium oxide, the use of shell powder in biomedicals or in cosmetics, the application of crushed shells as a liming agent for the neutralization of acidic soils and as a construction material for aggregate and mortar mixes [4]. Generally, for both environmental and economic aspects, these valorization strategies are exploited in areas where large amounts of shell waste are produced.

Shell can be used as calcium supplement for livestock for bone health and to increase the quality of eggshells. Several studies showed that shell-derived calcium carbonate can replaces limestone for this purpose [4,63]. However, today this type of valorization is limited to farms in proximity to shell waste production for both environmental and economic benefits mainly associated with distance and transportation costs. Moreover, the use of powdered waste shells for calcium supplementation has to be carefully evaluated for the safety issues due to possible trace amounts of heavy metals in the shell for bioaccumulation [4,10].

Another important use of shell involves the neutralization of acidic soils to reduce acidity and improve fertility. In this way, shell waste can replace the mined limestone that is usually used as liming agent for this treatment [64,65]. However, environmental concerns are associated with the high temperatures and operational procedures of the calcination of waste shells to obtain lime [4,10]. Moreover, a further environmental question is represented by the possible release of heavy metals due to the shell dissolution occurring in the neutralization reaction [10].

Many studies focused on the application of shells as materials used in construction, pigments, fillers, etc. showing that the market price of powdered CaCO_3_ ranges from 60 to 66 USD per ton for coarse particles to 14,000 USD per ton for ultrafine powder, usually applied to enhance the features of plastics and rubber [66]. In particular, the use of waste shells in concrete contributes in the management of the waste and in reducing natural limestone quarrying [4]. However, waste shells need pretreatment such as washing and calcination that use significant amounts of energy and resources, thus resulting in further environmental issues [10]. Moreover, the distance between shell waste sources and processing facilities to produce powdered CaCO_3_ is a key factor in shell valorization as construction materials [4].

Mollusk shells are biomaterials with features similar to those of human bones and teeth; therefore, this waste can be the starting source of CaO for the synthesis of calcium phosphate ceramics to be used for bone regeneration and dental applications [67,68]. Anyway, some shell waste-derived biomaterials have been currently produced only in laboratory scale and further investigation is necessary for the evaluation of their economic and environmental costs [4,10].

Finally, numerous studies showed that shell waste can be used as a precursor raw material for the production of low-cost HAP to be used for wastewater treatment and bone-related applications [10,69,70]. This route to produce HAP has numerous advantages over current production methods including low-cost precursors, simple synthesis procedure, low energy consumption, low temperature synthesis and high yield and purity, as well as the reduction of the environmental impact of disposed shell waste [10,69,71].

These few examples showed that shell waste valorization provides significant environmental and economic advantages and can contribute to reducing the impact of mining activities for the exploitation of natural resources such as limestone and marble.

## 4 Conclusions

This study showed that clam shell waste can be utilized as a raw material to sustainably synthesize a high-capacity adsorbent for removing Hg from polluted water. The Hg removal capacity of CSHAP has been evaluated in experiments carried out in batch at varying contact times and initial metal concentrations. The rapid adsorption process reached equilibrium in about 40 min where the Hg removal efficiency was about 95 %. The results of XRPD analysis indicated a gradual expansion of the unit cell of CSHAP as a function of the contact time due to the progressive incorporation of Hg ions into the crystalline structure. The removal of Hg resulted in a smooth morphology of the CSHAP grains, suggesting that dissolution-precipitation phenomena play a role in the overall removal mechanism. Moreover, new strong Hg peaks appeared in the EDS spectra after the reaction with the Hg containing solutions. The adsorption kinetics followed the pseudo-second-order model and the experimental data fitted better the Langmuir and Dubinin-Radushkevich isotherm models, showing a maximum adsorption capacity of 65.8 mg/g. The Hg removal mechanism involves rapid surface complexation on CSHAP grains, followed by a slower incorporation of Hg ions into the crystalline structure via ion exchange with Ca. The Hg removal capacity of CSHAP is remarkable when compared to other waste-derived adsorbents, with the primary advantage being the huge availability of the raw material along with the simplicity of the production process. This study highlighted that converting waste into an effective adsorbent for Hg remediation not only addresses water pollution but also contribute to the sustainable management of clam shell waste.

## Data availability

Data associated with this study has not been deposited into a publicly available repository. However, data are available from the corresponding author upon request.

## CRediT authorship contribution statement

**Silvano Mignardi:** Writing – review & editing, Supervision, Funding acquisition, Data curation, Conceptualization. **Emanuele Tocci:** Writing – original draft, Investigation, Formal analysis. **Laura Medeghini:** Writing – review & editing, Methodology, Investigation, Formal analysis, Data curation.

## Declaration of competing interest

The authors declare that they have no known competing financial interests or personal relationships that could have appeared to influence the work reported in this paper.

## References

[1] Zeng X., Ogunseitan O.A., Nakamura S., Suh S., Kral U., Li J., Geng Y. (2002). Reshaping global policies for circular economy. Circ. Econ..

[2] Lee M., Tsai W.S., Chen S.T. (2020). Reusing shell waste as a soil conditioner alternative? A comparative study of eggshell and oyster shell using a life cycle assessment approach. J. Clean. Prod..

[3] Ciccullo F., Cagliano R., Bartezzaghi G., Perego A. (2021). Implementing the circular economy paradigm in the agri-food supply chain: the role of food waste prevention technologies. Resour. Convers. Recycl..

[4] Morris J.P., Backeljau T., Chapellee G. (2019). Shells from aquaculture: a valuable biomaterial, not a nuisance waste product. Rev. Aquacult..

[5] Aljerf L., Aljerf N. (2023). Food products quality and nutrition in relation to public. Balancing health and disease. Prog. Nutr..

[6] FAO (2022).

[7] Hart A. (2020). Mini-review of waste shell-derived materials' applications. Waste Manag. Res..

[8] Habte L., Khan M.D., Shiferaw N., Farooq A., Lee M., Jung S., Ahn J.W. (2020). Synthesis, characterization and mechanism study of green aragonite crystals from waste biomaterials as calcium supplement. Sustainability.

[9] Vishwanatham K., Kumar B.S., Kumar D.R. (2024). Application of biotechnology in aquaculture. J. Adv. Zool..

[10] Topić Popović N., Lorencin V., Strunjak-Perović I., Čož-Rakovac R. (2023). Shell waste management and utilization: mitigating organic pollution and enhancing sustainability. Appl. Sci..

[11] Ferraz E., Gamelas J.A.F., Coroado J., Monteiro C., Rocha F. (2019). Recycling waste seashells to produce calcitic lime: characterization and wet slaking reactivity. Waste Biomass. Valor..

[12] Pranesh P., Gautam A. (2024). The shells of edible freshwater snails as a biosorbent for multi-metal ions from wastewater: implication in effluent purification and waste valorisation. Sep. Purif. Technol..

[13] Núñez D., Elgueta E., Varaprasad K., Oyarzún P. (2018). Hydroxyapatite nanocrystals synthesized from calcium rich bio-wastes. Mater. Lett..

[14] Mohd Pu’ad N.A.S.M., Koshy P., Abdullah H.Z., Idris M.I., Lee T.C. (2019). Syntheses of hydroxyapatite from natural sources. Heliyon.

[15] Zhou Z., Wang Y., Sun S., Wang Y., Xu L. (2022). Preparation of PVA/waste oyster shell powder composite as an efficient adsorbent of heavy metals from wastewater. Heliyon.

[16] Quina M.J., Soares M.A.R., Quinta-Ferreira R. (2017). Applications of industrial eggshell as a valuable anthropogenic resource. Resour. Conserv. Recycl..

[17] Cao X., Ma L.Q., Chen M., Singh S.P., Harris W.G. (2002). Impacts of phosphate amendments on lead biogeochemistry at a contaminated site. Environ. Sci. Technol..

[18] Smičiklas I., Onjia A., Raičević S., Janaćković Ð., Mitrić M. (2008). Factors influencing the removal of divalent cations by hydroxyapatite. J. Hazard Mater..

[19] Nzihou A., Sharrock P. (2010). Role of phosphate in the remediation and reuse of heavy metal polluted wastes and sites. Waste Biomass Valor.

[20] De Angelis G., Medeghini L., Conte A.M., Mignardi S. (2017). Recycling of eggshell waste into low-cost adsorbent for Ni removal from wastewater. J. Clean. Prod..

[21] Ibrahim M., Labaki M., Giraudon J.M., Lamonier J.F. (2019). Hydroxyapatite, a multifunctional material for air, water and soil pollution control: a review. J. Hazard Mater..

[22] Foroutan R., Peighambardoust S.J., Ahmadi A., Akbari A., Farjadfard S., Ramavandi B. (2021). Adsorption mercury, cobalt, and nickel with a reclaimable and magnetic composite of hydroxyapatite/Fe_3_O_4_/polydopamine. J. Environ. Chem. Eng..

[23] Mignardi S., Archilletti L., Medeghini L., De Vito C. (2020). Valorization of eggshell biowaste for sustainable environmental remediation. Sci. Rep..

[24] Pérez-Marín A.B., Zapata V.M., Ortuño J.F., Aguilar M., Sáez J., Lloréns M. (2007). Removal of Cd from aqueous solutions by adsorption onto orange waste. J. Hazard Mater..

[25] Selin H., Keane S.E., Wang S., Selin N.E., Davis K., Bally D. (2018). Linking science and policy to support the implementation of the Minamata Convention on Mercury. Ambio.

[26] Rani L., Srivastav A.L., Kaushal J., X.C. Nguyen X.C. (2022). Recent advances in nanomaterial developments for efficient removal of Hg(II) from water. Environ. Sci. Pollut. Res..

[27] Liu S., Wang X., Guo G., Yan Z. (2021). Status and environmental management of soil mercury pollution in China: a review. J. Environ. Manag..

[28] Obrist D., Kirk J.L., Zhang L., Sunderland E.M., Jiskra M., Selin N.E. (2018). A review of global environmental mercury processes in response to human and natural perturbations: changes of emissions, climate, and land use. Ambio.

[29] Camargo C.L.M., Salim V.M.M., Tavares F.W., Solange de Resende N. (2018). Phenomenological modeling for elemental mercury capture on hydroxyapatite-based adsorbents: an experimental validation. Fuel.

[30] Monteagudo J.M., Ortiz M.J. (2000). Removal of inorganic mercury from mine wastewater by ion exchange. J. Chem. Technol. Biotechnol..

[31] Rimondi V., Gray J.E., Costagliola P., Vaselli O., Lattanzi P. (2012). Concentration, distribution, and translocation of mercury and methylmercury in mine-waste, sediment, soil, water, and fish collected near the Abbadia San Salvatore mercury mine, Monte Amiata district, Italy. Sci. Total Environ..

[32] Aljerf L. (2018). High-efficiency extraction of bromocresol purple dye and heavy metals as chromium from industrial effluent by adsorption onto a modified surface of zeolite: kinetics and equilibrium study. J. Environ. Manag..

[33] Langmuir I. (1918). Adsorption of gases on plain surfaces of glass, mica and platinum. J. Am. Chem. Soc..

[34] Freundlich H.M.F. (1906). Over the adsorption in solution. J. Phys. Chem..

[35] Temkin M.I., Pyzhev V. (1940). Recent modifications to Langmuir isotherms. Acta Phys. Chim. USSR.

[36] Dubinin M.M., Radushkevich L.V. (1947). The equation of the characteristic curve of the activated charcoal. Proc. Acad. Sci. USSR Phys. Chem. Sect..

[37] Majd M.M., Kordzadeh-Kermani V., Ghalandari V., Askari A., Sillanpää M. (2022). Adsorption isotherm models: a comprehensive and systematic review (2010− 2020). Sci. Total Environ..

[38] Foo K.Y., Hameed B.H. (2010). Insights into the modeling of adsorption isotherm systems. Chem. Eng. J..

[39] Amer M.W., Ahmad R.A., Awwad A.M. (2015). Biosorption of Cu(II), Ni(II), Zn(II) and Pb(II) ions from aqueous solution by *Sophora japonica* pods powder. Int. J. Ind. Chem..

[40] Lagergren S. (1898). About the theory of so-called adsorption of soluble substances, K. Sven. Vetenskapsakad. Handl..

[41] Ho Y.S., McKay G. (2003). Sorption of dyes and copper onto biosorbents. Process Biochem..

[42] Weber W.J., Morris J.C. (1963). Kinetics of adsorption on carbon from solution. J. Sanit. Eng. Div. Am. Soc. Civ. Eng..

[43] Largitte L., Pasquier R. (2016). A review of the kinetics adsorption models and their application to the adsorption of lead by an activated carbon. Chem. Eng. Res. Des..

[44] de Resende N.S., Camargo C.L.M., Reis P.C., Perez C.A.C., Salim V.M.M. (2019). Mechanisms of mercury removal from aqueous solution by high-fixation hydroxyapatite sorbents. Int. J. Sci. Environ. Technol..

[45] Aljerf L., Choukaife A.E. (2017). Hydroxyapatite and fluoroapatite behavior with pH change. Int. Med. J..

[46] Destainville A., Champion E., Bernache-Assollant D., Laborde E. (2003). Synthesis, characterization and thermal behavior of apatitic tricalcium phosphate. Mater. Chem. Phys..

[47] Meejoo S., Maneeprakorn W., Winotai P. (2006). Phase and thermal stability of nanocrystalline hydroxyapatite prepared via microwave heating. Thermochim. Acta.

[48] Rehman I., Bonfield W.J.J. (1997). Characterization of hydroxyapatite and carbonated apatite by photo acoustic FTIR spectroscopy. J. Mater. Sci. Mater. Med..

[49] Choukaife A.E., Aljerf L. (2017). A descriptive study - in vitro: new validated method for checking Hap and Fap behaviours. Int. Med. J..

[50] Tang J., Li Y., Wang X., Daroch M. (2017). Effective adsorption of aqueous Pb^2+^ by dried biomass of *Landoltia punctata* and *Spirodela polyrhiza*. J. Clean. Prod..

[51] Corami A., Mignardi S., Ferrini V. (2008). Cadmium removal from single- and multimetal (Cd+Pb+Zn+Cu) solutions by sorption on hydroxyapatite. J. Colloid Interface Sci..

[52] Zhang Y., Xia M., Wang F., Ma J. (2021). Experimental and theoretical study on the adsorption mechanism of Amino trimethylphosphate (ATMP) functionalized hydroxyapatite on Pb (II) and Cd (II). Colloid Surf. A - Physicochem. Eng. Asp..

[53] Xu D., Tan X.L., Chen C.L., Wang X.K. (2008). Adsorption of Pb(II) from aqueous solution to MX-80 bentonite: effect of pH, ionic strength, foreign ions and temperature. Appl. Clay Sci..

[54] Eren E., Afsin B., Onal Y. (2009). Removal of lead ions by acid activated and manganese oxide-coated bentonite. J. Hazard Mater..

[55] Özcan A., Safa Özcan A., Tunali S., Akar T., Kiran I. (2005). Determination of the equilibrium, kinetic and thermodynamic parameters of adsorption of copper(II) ions onto seeds of *Capsicum annuum*. J. Hazard Mater..

[56] Mariana M., Mistar E.M., Syabriyana M., Zulkipli A.S., Aswita D., Alfatah T. (2022). Properties and adsorptive performance of candlenut shell and its porous charcoals for aqueous mercury (II) removal. Bioresour. Technol. Rep..

[57] Qian X., Wang R., Zhang Q., Sun Y., Li W., Zhang L., Qu B. (2022). A delicate method for the synthesis of high-efficiency Hg (II) the adsorbents based on biochar from corn straw biogas residue. J. Clean. Prod..

[58] Hassan S.S.M., Awwad N.S., Aboterika A.H.A. (2008). Removal of mercury(II) from wastewater using camel bone charcoal. J. Hazard Mater..

[59] Giraldo S., Robles I., Ramirez A., Flórez E., Acelas N. (2020). N., Mercury removal from wastewater using agroindustrial waste adsorbents. SN Appl. Sci..

[60] Liu Z., Sun Y., Xu X., Qu J., Qu B. (2020). Adsorption of Hg (II) in an aqueous solution by activated carbon prepared from rice husk using KOH activation. ACS Omega.

[61] Guo Y.F., Wang Z., Zhou X.J., Bai R.B. (2017). Removal of mercury (II) from aqueous solution with three commercial raw activated carbons. Res. Chem. Intermed..

[62] Sricharoen P., Limchoowong N., Nuengmatcha P., Chanthai S. (2020). Ultrasonic-assisted recycling of Nile tilapia fish scale biowaste into low-cost nano-hydroxyapatite: ultrasonic-assisted adsorption for Hg^2+^ removal from aqueous solution followed by “turn-off” fluorescent sensor based on Hg^2+^-graphene quantum dots. Ultrason. Sonochem..

[63] Çatli A.U., Bozkurt M., Küçükyılmaz K., Çınar M., Bintaş E., Çöven F., Atik H. (2012). Performance and egg quality of aged laying hens fed diets supplemented with meat and bone meal or oyster shell meal. S. Afr. J. Anim. Sci..

[64] Garrido-Rodríguez B., Fernández-Calviño D., Nóvoa Muñoz J.C., Arias-Estévez M., Díaz-Raviña M., Álvarez-Rodríguez E., Fernández-Sanjurjo M.J., Núñez-Delgado A. (2013). pH-dependent copper release in acid soils treated with crushed mussel shell. Int. J. Environ. Sci. Technol..

[65] Hamilton S.K., Kurzman A.L., Arango C., Jin L., Robertson G.P. (2007). Evidence for carbon sequestration by agricultural liming. Global Biogeochem. Cycles.

[66] Yan N., Chen X. (2015). Don't waste seafood waste. Nature.

[67] Vecchio K.S., Zhang X., Massie J.B., Wang M., Kim C.W. (2007). Conversion of bulk seashells to biocompatible hydroxyapatite for bone implants. Acta Biomater..

[68] Wu S.C., Hsu H.C., Wu Y.N., Ho W. (2011). Hydroxyapatite synthesized from oyster shell powders by ball milling and heat treatment. Mater. Char..

[69] Luo H., Huang G., Fu X., Liu X., Zheng D., Peng J., Zhang K., Huang B., Fan L., Chen F., Sun X. (2013). Waste oyster shell as a kind of active filler to treat the combined waste water at an estuary. J. Environ. Sci..

[70] Wang Z., Dong J., Liu L., Zhu G., Liu C. (2013). Screening of phosphate-removing substrates for use in constructed wetlands treating swine wastewater. Ecol. Eng..

[71] Summa D., Lanzoni M., Castaldelli G., Fano E.A., Tamburini E. (2022). Trends and opportunities of bivalve shells' waste valorization in a prospect of circular blue bioeconomy. Resources.

